# Value of electrophysiological indicators in differential diagnosis of parkinson's disease and multiple system atrophy

**DOI:** 10.1186/s12883-023-03131-8

**Published:** 2023-03-02

**Authors:** Wangwang Hu, Yifan Cheng, Jie Pan, Xun Wang, Shaojing Li, Zijian Fan, Bei Shao, Xiaoting Niu

**Affiliations:** 1 Department of Rehabilitation Medicine, Ningbo Medical Center Li Huili Hospital, Zhejiang 315000 Ningbo, China; 2 Center for Rehabilitation Medicine, Department of Neurology, Zhejiang Provincial People’s Hospital, Affiliated People’s Hospital, Hangzhou, 310014 Zhejiang China; 3 Department of Neurology, The First Affiliated Hospital of Wenzhou Medical University, Wenzhou, 325000 Zhejiang China; 4 Department of Neurology, Taizhou Central Hospital, Taizhou, 317700 Zhejiang China

**Keywords:** Multiple system atrophy, Parkinson's disease, External anal sphincter electromyography, Bulbocavernosus reflex

## Abstract

**Background:**

We evaluated the value of electrophysiological indicators by external anal sphincter electromyography (EAS-EMG), sympathetic skin response (SSR), R-R interval variation (RRIV), and Bulbocavernosus Reflex (BCR) in differential diagnosis of multiple system atrophy (MSA) and Parkinson’s disease (PD).

**Methods:**

A total of 41 patients with MSA and 32 patients with PD were enrolled. The electrophysiological changes of autonomic dysfunction were assessed with BCR, EAS-EMG, SSR, and RRIV, and the abnormal rate of each indicator was calculated. The diagnostic value of each indicator was analyzed with ROC curve.

**Results:**

The incidence rate of autonomic dysfunction in MSA group was significantly higher than that in PD group (*p* < 0.05). The abnormal rates of BCR and EAS-EMG indicators in MSA group were higher than those in PD group (*p* < 0.05). The abnormal rates of SSR and RRIV indicators in MSA group and PD group were high; however, there was no significant difference between MSA and PD groups (*p* > 0.05). The sensitivity of BCR combined with EAS-EMG indicators in differential diagnosis of MSA and PD were 92.3% in males and 86.7% in females, respectively, and the specificity was 72.7% in males and 90% in females, respectively.

**Conclusions:**

Combined analysis of BCR and EAS-EMG has high sensitivity and specificity for differential diagnosis of MSA and PD.

## Background

Parkinson’s disease (PD) and multiple system atrophy (MSA) are neurodegenerative disorders pathologically characterized by the accumulation of insoluble α-synuclein protein in the nervous system [1]. Clinically, in addition to having overlapping motor features, patients with PD and MSA commonly experience non-motor symptoms. Many MSA patients have complex and atypical early symptoms, and even respond well to levodopa, especially MSA-P type. It is difficult to distinguish MSA from PD based on motor symptoms alone [2]. Therefore, non-motor symptoms have attracted increasing attention in the differential diagnosis between MSA and PD.

MSA is pathologically characterized by eosinophilic Lewy inclusion in the cytoplasm of oligodendrocytes, with abnormal accumulation of α-synuclein as the core component. Eosinophilic Lewy inclusion is more widely distributed and simultaneously disrupts multiple neurotransmitter circuits such as dopamine, serotonin, acetylcholine, and norepinephrine. Therefore, patients with MSA had more extensive and severe non-motor symptoms than those with PD [3]. Non-motor symptoms in MSA are mainly manifested as autonomic dysfunction, such as urination disturbance, sexual dysfunction, blood pressure variation, etc., which can appear earlier than motor symptoms. Moreover, autonomic dysfunction was presented as the first symptom in more than 50% of MSA [4]. Therefore, objective assessment of autonomic dysfunction is importance for the diagnosis and differentiation of MSA and PD.

It has been shown that external anal sphincter electromyography (EAS-EMG), sympathetic skin response (SSR), R-R interval variation (RRIV), and Bulbocavernosus Reflex (BCR) can be used to evaluate autonomic nerve involvement [1, 5]. Although both EAS-EMG and BCR have high sensitivity and specificity for the differential diagnosis of MSA, the positive rates of diagnosis across various centers are different due to the limitations and operational errors of each examination. Herein, we first evaluated the autonomic dysfunction of MSA and PD patients with EAS-EMG, SSR, RRIV and BCR. Then, we compared the diagnostic value of these electrophysiological examination methods for differential diagnosis of MSA and PD. Our findings may help improve the diagnosis rate and early diagnosis of MSA and PD.

## Methods

### Ethics

This study was approved by the Scientific Research Management Ethics Committee of the First Affiliated Hospital of Wenzhou Medical University (Approval No.: 2016–179). All methods were carried out in accordance with the Declaration of Helsinki. Written informed consent was obtained from the family members of all participants.

### Patients

A total of 41 patients with MSA and 32 patients with PD were consecutively recruited at the First Affiliated Hospital of Wenzhou Medical University from December 2017 to December 2021. MSA was diagnosed according to the 2017 edition of Expert Consensus on Diagnosis Criteria for Multiple System Atrophy in China [5], and PD was diagnosed according to the Movement Disorders Society clinical diagnostic criteria [6]. Exclusion criteria: 1) Patients with history of spinal cord lesions, lumbosacral radiculopathy, diabetic peripheral neuropathy, anorectal and pelvic floor diseases were excluded; 2) male patients with history of prostate disease were also excluded.

### Data collection

The basic clinical information, including age, sex, disease course, and vaginal delivery (for females), were collected. The autonomic dysfunction was evaluated with SCOPA-AUT (Scales for Outcomes in Parkinson's Disease-Autonomic questionnaire) by a qualified neurologist. The symptoms of autonomic dysfunction, such as orthostatic hypotension, urinary incontinence, incomplete bladder emptying, frequent urination, constipation, and male sexual dysfunction, were also recorded.

### Electrophysiological examination

EAS-EMG [7], BCR [8], SSR, and RRIV tests [9] were performed by using Keypoint electromyography/evoked potential instrument (Vidi, Denmark) with the cooperation of experienced neurologists and EMG technicians. The average latency of the left and right sides of the BCR, the average duration, average amplitude, polyphase wave ratio and satellite potential occurrence rate of EAS-EMG Motor Unit Potentials (MUPs), the latency and amplitude of SSR, and RRIV of deep and calm breathing were recorded.

### Statistical analysis

Statistical analysis was performed using SPSS 25.0(IBM SPSS, USA). Kolmogorov–Smirnov (K-S) normality test was performed on all data. Continuous variables with normal distribution were expressed as mean ± standard deviation (SD) and compared with independent t-test. Continuous variables with non-normal distribution were expressed as median and interquartile range (IQR) and analyzed with Kruskal–Wallis H or Mann–Whitney U tests. Comparisons of categorical variables were conducted by using chi-square test, which were expressed as frequencies and percentages. The receiver operating characteristic curve (ROC curve) was plotted with the clinical diagnosis as the state variable and the area under the curve (AUC) was calculated. The maximum critical value of Youden index (Youden index = sensitivity + specificity—1) was used as a diagnostic predictive value. The difference was considered statistically significant at *P* < 0.05. Since the parameters of BCR and EAS-EMG are affected by gender [7, 8], male and female patients were independently compared in this study.

## Results

### Baseline clinical information of patients

The baseline clinical information of patients was shown in Table 1. In detail, a total of 41 patients with clinically diagnosed MSA, including 26 males and 15 females, were included. Their average age was (62.8 ± 8.9) years and their average disease duration was (3.5 ± 2.4) years. A total of 32 patients with PD, including 22 males and 10 females, were included, with an average age of (66.3 ± 10.6) years and average disease duration of (4.7 ± 4.1) (0.3–20) years. According to Hoehn-Yahr staging method, there were 1 case with Hoehn-Yahr stage I, 8 cases with Hoehn-Yahr stage II, 9 cases with Hoehn-Yahr stage III, 12 cases with Hoehn-Yahr stage IV and 2 cases with Hoehn-Yahr stage V. For treatment, 1 case did not receive PD drug treatment, 11 cases received compound levodopa monotherapy, 2 cases received MAO-BI monotherapy, and 1 case received amantadine monotherapy. The other 17 patients received 2–4 kinds of PD drugs. There was no significant difference in sex ratio between MSA group and PD group (*p* = 0.634). There was no significant difference in terms of the course of disease and age between MSA group and PD group, MSA males and PD males, as well as MSA females and PD females (all *p* > 0.05). There was no significant difference in the proportion of females with or without vaginal delivery between MSA and PD (*p* = 0.543). The SCOPA-AUT score, and, the incidence of orthostatic hypotension, urinary incontinence, and male sexual dysfunction, in the MSA group were significantly higher than those in PD group (all *p* < 0.05, Table 1).

**Table 1:** Baseline characteristics and symptoms of autonomic dysfunction in patients with MSA and PD

	MSA			PD		
		Male	Female		Male	Female
No	41	26	15	32	22	10
Age (years)a	62.6 ± 8.9	64.3 ± 8.7	59.8 ± 8.8	66.3 ± 10.6	66.2 ± 12.1	66.3 ± 6.5
Clinical disease course (years)^a^	3.5 ± 2.4	3.4 ± 2.4	3.6 ± 2.6	4.7 ± 4.1	4.9 ± 4.7	4.4 ± 2.3
Vaginal delivery ^b^			14			8
SCOPA-AUT score ^a^	30.4 ± 7.2#	30.8 ± 6.8@	29.7 ± 8.0&	16.5 ± 7.1	14.6 ± 5.8	20.5 ± 8.3
Orthostatic hypotension (%)^c^	31.7 (13/41)*	26.9 (7/26)*	40.0(6/15)*	6.3 (2/32)	4.5 (1/22)	10.0(1/10)
Urinary incontinence (%)^c^	53.7 (22/41)$	50.0(13/26)$	60.0(9/15)$	28.1 (9/32)	27.3(6/22)	30.0(3/10)
Incomplete bladder emptying (%)^c^	61.0 (25/41)	61.5 (16/26)	60.0(9/15)	37.5 (12/32)	36.4(8/22)	40.0(4/10)
Frequent urination (%)^c^	39.0 (16/41)	42.3(11/26)	33.3 (5/15)	56.3 (18/32)	59.1(13/22)	50.0 (5/10)
Constipation(%)^c^	82.9 (34/41)	80.8 (21/26)	86.7(13/15)	68.8 (22/32)	72.7(16/22)	60.0(6/10)
Male sexual dysfunction (%)^c^	88.5 (23/26)#			36.4 (8/22)		

a: t test; b: Fisher's exact test; c: Pearson X2 test

^#^: compared with PD group, *p* < 0.001; @: compared with PD male group, *p* < 0.001; &: compared with PD female group compared, *p* < 0.05; *: compared with PD group, *p* < 0.01; $: compared with PD group, *p* < 0.05

### Comparison of BCR latency and abnormal rate

**Fig. 1:**
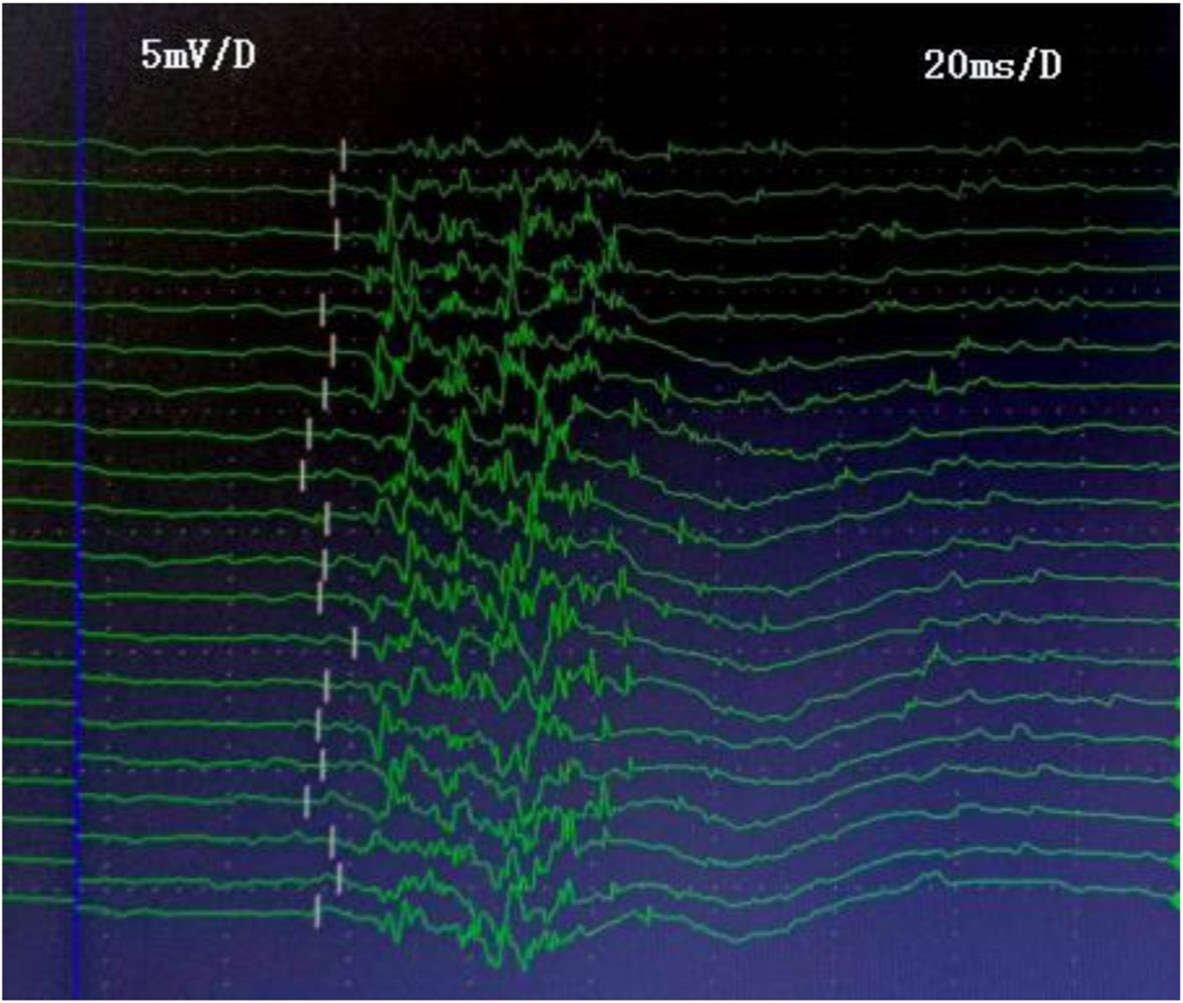
BCR test in a 59-year-old male patient with PD. The mean BCR latency was 60.4 ms. It can be seen that the BCR latency was prolonged in this patient

The image of typical case with abnormal BCR is shown in Fig. 1. Comparison of BCR latency and abnormal rate between groups was showed in Table 2. The BCR latency of male and female MSA patients was significantly longer than that of PD patients of the corresponding sex (both *p* < 0.05). Similarly, the abnormal rate of BCR in male and female MSA patients was significantly higher than that in PD patients of the corresponding sex (*p* < 0.05). However, there was no significant difference in BCR latency between left and right sides (*p* > 0.05).

**Table 2:** Comparison of BCR latency and abnormal rate between MSA and PD patients

	Males			Females			Total
	Abnormal rate (%)	BCR latency (ms)		Abnormal rate (%)	BCR latency (ms)		Abnormal rate (%)
		Left	Right		Left	Right	
MSA	69.2 (18/26)	49.83 ± 12.62	49.22 ± 12.82	60.0 (9/15)	60.54 ± 9.48	60.03 ± 8.99	65.9(27/41)
PD	27.3 (6/22)	34.70 ± 7.29	34.94 ± 7.64	20.0 (2/10)	47.81 ± 8.15	48.52 ± 9.29	25(8/32)
P	0.004	0.000	0.000	0.048	0.008	0.017	0.005

### Comparison of EAS-EMG parameters

**Fig. 2:**
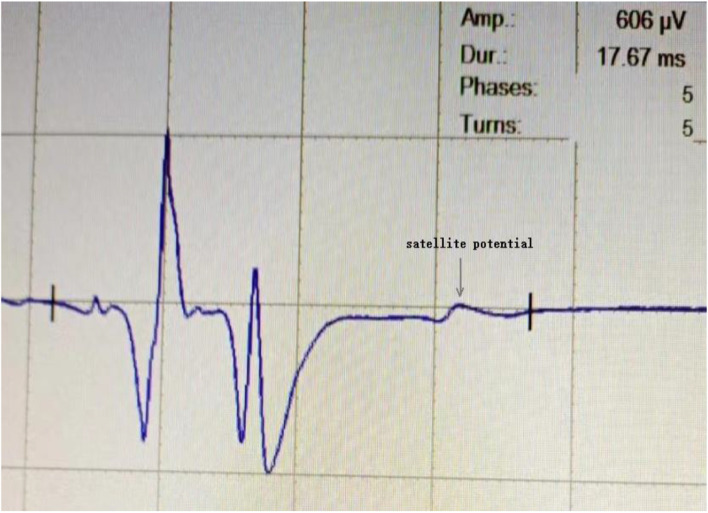
EAS-EMG test in a 65-year-old female patient with MSA. EAS-EMG latency was 17.67 ms. EAS-EMG amplitude was 606 μV. In this patient, EAS-EMG latency was significantly prolonged, and satellite potential was observed

The image of typical case with abnormal EAS-EMG is shown in Fig. 2. As shown in Table 3, the average duration of MUPs, the proportion of polyphase waves, and the occurrence rate of satellite potentials in the MSA group of males and females were significantly higher than those of the PD group (*p* < 0.05). There were 38 MSA patients (24 males and 14 females) with abnormal EAS-EMG parameters, with an abnormal rate of 92.7% (38/41). However, only 9 PD patients (6 males and 3 females) had abnormal EAS-EMG parameters, with an abnormal rate of 28.1% (9/32). There were significant differences in the abnormal rate of EAS-EMG between MSA vs. PD, MSA male vs. PD male, and, MSA female vs. PD female (all *p* < 0.05).

**Table 3:** Comparison of EAS-EMG parameters between MSA and PD groups

	Males			Females		
	MSA	PD	P	MSA	PD	P
Number of cases	26	22	-	15	10	-
Average duration time (ms)	12.15 ± 1.74	8.59 ± 1.74	0.000	11.80 ± 1.57	9.77 ± 1.37	0.003
Average amplitude (μV)	607.52 ± 156.47	577.07 ± 145.22	0.491	641.97 ± 122.59	528.85 ± 178.16	0.072
Polyphase Wave Ratio (%)	48.04 ± 13.80	34.09 ± 16.00	0.002	51.78 ± 19.02	29.51 ± 12.16	0.002
Satellite potential occurrence rate (%)	92.3(24/26)	27.3(6/22)	0.000	93.3 (14/15)	30 (3/10)	0.001

### Comparison of SSR and RRIV parameters

There was also no significant difference in SSR latency and amplitude between MSA and PD groups (all *p* > 0.05) (Table 4). Similarly, the abnormal SSR rates in MSA group and PD group were 97.6% (40/41) and 90.6% (29/32), respectively, without significant difference (*p* > 0.05). The RRIV measured during calm breathing and deep breathing was not significantly different between MSA and PD groups (all *p* > 0.05) (Table 4). The abnormal rate of RRIV in MSA group was 95.1% (39/41), which was not significantly different from that in PD group (84.4% (27/32)) (*p* > 0.05).

**Table 4:** Comparison of SSR and RRIV results between MSA and PD groups

	SSR										RRIV		
	Upper extremity latency (ms)		Lower extremity latency (ms)		Upper extremity amplitude (mV)			Lower extremity amplitude (mV)		Abnormal rate	RRIV during calm breathing (%)	RRIV during deep breathing (%)	Abnormal rate (%)
	Left	Right	Left	Right	Left	Right	Left		Right				
MSA	1597.81 ± 186.26	1628.47 ± 145.76	2085.54 ± 222.35	2210.90 ± 268.5	0.68 (0.41, 0.68)	0.62 (0.39, 0.85)	0.44 (0.30, 0.91)		0.45 (0.25, 0.69)	97.6 (40/41)	7.62 ± 4.71	11.89 ± 6.77	95.1 (39/41)
PD	1627.13 ± 264.80	1604.88 ± 301.25	2097.21 ± 234.50	2107.54 ± 257.39	0.71 (0.49, 1.48)	0.84 (0.38, 1.43)	0.66 (0.38, 0.84)		0.69 (0.45, 0.99)	90.6% (29/32)	9.22 ± 4.45	12.70 ± 6.08	84.4 (27/32)
P	0.720	0.764	0.896	0.359	0.926	0.523	0.488		0.056	0.439	0.145	0.595	0.251

### ROC analysis

**Fig. 3:**
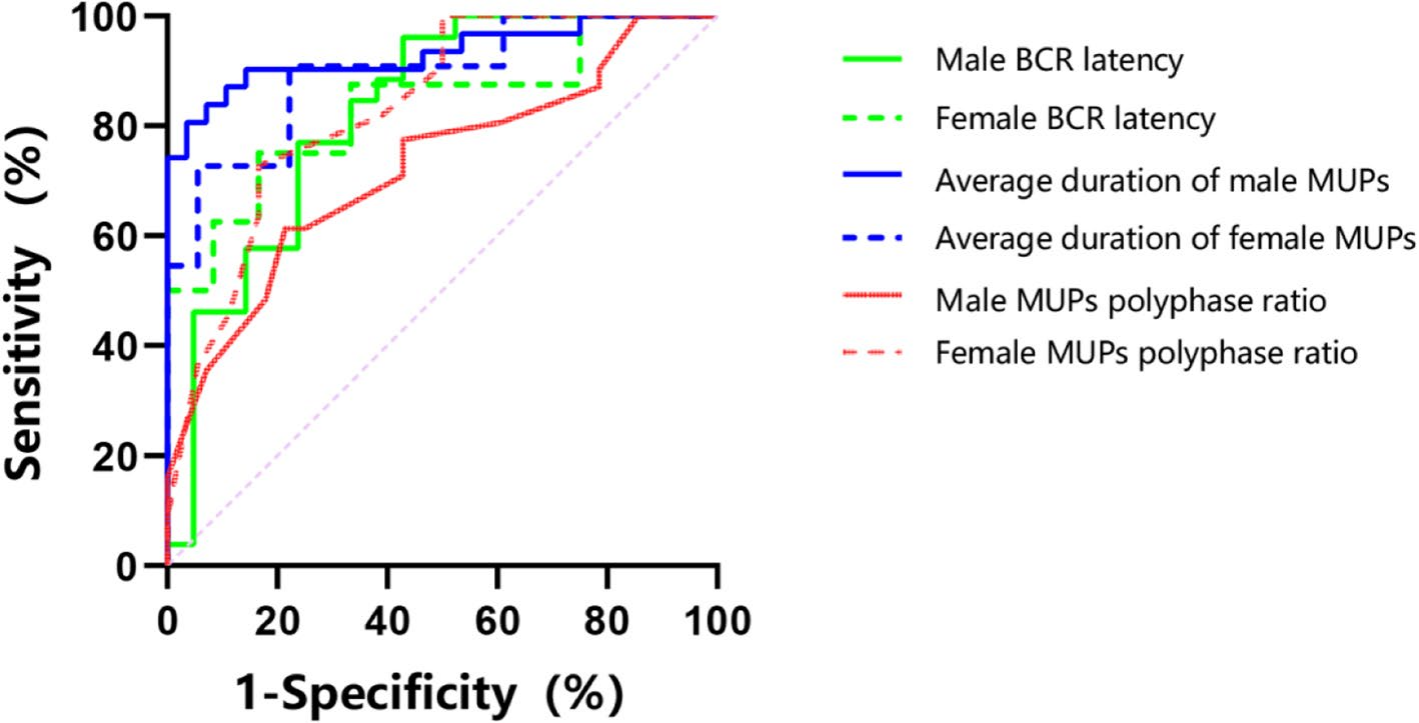
ROC curve analysis of each neurophysiological index of BCR and EAS-EMG in the identification of MSA and PD

ROC curve was used to analyze the diagnostic value of each electrophysiological indicator for discriminating MSA from PD (Table 5 and Fig. 3). The AUC of BCR latency, EAS-EMG MUPs mean duration, polyphasicity ratio, and satellite potential between male MSA and PD patients was 0.860, 0.951, 0.740, and 0.851, respectively. The critical values were 35.75 ms, 10.38 ms and 35.75%, respectively. The sensitivity of the differential diagnosis was 89.5%, 92.3%, and 80.8%, and the specificity was 68.4%, 86.4%, and 59.1%, respectively. For differential diagnosis between female MSA and PD patients, the AUC of BCR latency, the EAS-EMG MUPs mean duration, polyphasicity ratio, and satellite potential was 0.840, 0.843, 0.833, and 0.808, respectively. The critical values were 51.23 ms, 10.78 ms and 37.5%, with high sensitivity and specificity. The AUC was all > 0.7, indicating that these electrophysiological indicators have certain reference value for the differential diagnosis of multisystem atrophy and Parkinson's disease. Among male patients, the sensitivity and specificity of BCR combined with EAS-EMG in discriminating MSA from PD were 92.3% and 72.7%, respectively. Among female patients, the sensitivity was 86.7% and the specificity was 90%. Differential diagnosis between MSA and PD may be effectively achieved based on the diagnostic cut-off values of these electrophysiological tests.

**Table 5:** ROC curve analysis of each neurophysiological index in the identification of MSA and PD

	AUC	Cutoff value	Sensitivity (%)	Specificity (%)	Standard error	P	95%CI
Male BCR latency (ms)	0.860	35.75	89.5	68.4	0.058	0.000	0.746 ~ 0.974
Female BCR latency (ms)	0.840	51.23	88.9	66.7	0.096	0.015	0.650 ~ 1.000
Average duration of male MUPs (ms)	0.951	10.38	92.3	86.4	0.033	0.000	0.887 ~ 1.000
Average duration of female MUPs (ms)	0.843	10.78	86.7	80.0	0.085	0.004	0.677 ~ 1.000
Male MUPs polyphase ratio (%)	0.740	35.75	80.8	59.1	0.072	0.005	0.598 ~ 0.881
Female MUPs polyphase ratio (%)	0.833	37.5	83.3	72.7	0.075	0.003	0.687 ~ 0.98
Male satellite potential (%)	0.851				0.053	0.000	0.747 ~ 0.956
Female satellite potential (%)	0.808				0.092	0.006	0.628 ~ 0.988

## Discussion

It is difficult to differentiate MSA and PD at the early stage only based on motor symptoms [4]. Both of MSA and PD can present autonomic nerve involvement symptoms to varying degrees. Objective assessment of autonomic nerve function impairment is of great significance in diagnosing and differentiating MSA and PD. In this study, we showed that the SCOPA-AUT score of the MSA group was significantly higher than that of the PD group, suggesting that the autonomic dysfunction of the MSA patients was more obvious than that of the PD patients, especially orthostatic hypotension, urinary incontinence, and male sexual dysfunction, which was consistent with previous study [5]. Moreover, we found that PD patients also have symptoms of different degrees of autonomic nerve involvement. Therefore, symptoms of autonomic nerve involvement cannot be used to distinguish MSA from PD. EAS-EMG, BCR, SSR and RRIV are electrophysiological indicators that can objectively reflect the autonomic nerve involvement, and their performance in MSA and PD is different [10–14]. However, there is no systematic and comprehensive report on which electrophysiological indicators are better in identifying MSA and PD. On the other hand, because each electrophysiological detection method has certain operating errors, the combination of multiple electrophysiological indicators may better improve the accuracy of the differential diagnosis between MSA and PD. In this study, for the first time, BCR, EAS-EMG, SSR, and RRIV were combined to discriminate MSA from PD. The above detection methods are all objective electrophysiological methods for evaluating autonomic dysfunction related to urination and defecation disorders. BCR refers to the contraction of the bulbocavernosus muscle by stimulation of the pudendal nerve. The BCR follows the sacral reflex arc, and its latency reflects the integrity of pudendal sensory afferent nerve, sacral S2-4, and efferent motor fibers [10]. Over the past 20 years, BCR has been used for the diagnosis of various neurogenic disorders, including neurogenic impotence, diabetic neurogenic bladder, spinal shock, and cauda equina syndrome [15, 16]. Cai et al. [17] used BCR for the differential diagnosis of PD and MSA for the first time, and found that BCR latency could be used as an electrophysiological index to differentiate MSA from PD. We obtained consistent results in this study. However, compared with PD patients, MSA patients had significantly longer BCR latency, indicating more severe damage to the sacral reflex arc with predominantly peripheral neuropathy, which was consistent with the difference in autonomic function scale scores.

One of the pathological features of MSA is neuronal loss in the nucleus of Onuf in the anterior horn of the sacral cord [18]. The nucleus of Onuf innervates the urethral sphincter and external anal sphincter through the pudendal nerve and plays an important role in the control of urination and defecation [10]. The neurogenic changes of EAS-EMG are manifested as prolonged duration, and, increased amplitude, polyphasic wave proportion, and satellite potential occurrence rate, which can indirect reflect the degeneration of the nucleus of Onuf [10, 17]. A large number of studies [11, 19] have shown that EAS-EMG can be used as a specific tool to distinguish PD from MSA, but some study has raised controversy about its diagnostic value [20]. Herein, this study showed that the abnormal rate of EAS-EMG in MSA patients was 92.7%, while the abnormal rate of BCR was 65.9%. The nerve center of the bulbocavernosus muscle is located in the dorsomedial nucleus of the Onuf nucleus [21]. It is speculated that the reason for the inconsistency of EAS-EMG and BCR may be that the Onuf nucleus is only a site on the reflex arc, and that the Onuf nucleus lesions in the early stage of MSA may be earlier. In pudendal neuropathy, when the degree of autonomic neuropathy is mild, only the nucleus of Onuf is involved, which may not cause abnormal nerve conduction [22]. In PD patients, the consistency between BCR and EAS-EMG was high, and it is speculated that the main cause of urinary and defecation disorders in PD patients may be the damage of the pudendal nerve.

SSR is an objective and sensitive index for evaluating the function of sympathetic postganglionic fibers, which is related to the activity of sweat glands [23]. SSR is widely used in the evaluation of autonomic nerve function in peripheral neuropathy, chronic renal failure, leprosy and central nervous system diseases (such as amyotrophic lateral sclerosis, PD, MSA, etc.) [24]. Abnormal SSR in PD patients is related to disease course and disease severity [12]. In this study, there was no significant difference in the abnormal rate of SSR between MSA and PD patients, suggesting that SSR has little value in the differential diagnosis of MSA and PD. This may also be related to the longer disease duration of the included PD cases.

RRIV reflects vagus nerve function [13]. RRIV is highly sensitive for detecting subclinical cardiovascular autonomic neuropathy but lacks specificity. In this study, we found that both MSA and PD patients had a higher proportion of abnormal RRIV, which was consistent with previous study [14].

The ROC curve was used to analyze the differential diagnostic value of each electrophysiological index in distinguishing MSA and PD. The BCR latency, the average duration of EAS-EMG MUPs, the heterogeneity ratio and the incidence of satellite potentials had high value of sensitivity and specificity, with AUC > 0.7, indicating their value for the differential diagnosis of MSA and PD. Based on the diagnostic cut-off values of these electrophysiological indictors, MSA and PD may be differentiated quickly, simply, and accurately.

## Conclusion

In conclusion, we showed that the SCOPA-AUT score of the MSA group was significantly higher than that of the PD group, suggesting that the autonomic dysfunction of the MSA patients was more obvious than that of the PD patients. The latency of BCR, the average duration of EAS-EMG MUPs, the proportion of polyphase waves, and the occurrence rate of satellite potentials in MSA patients were higher than those in PD patients. The corresponding areas of these indicators under the ROC curve were all > 0.7, suggesting that BCR and EAS-EMG MUPs may have good differential diagnostic value for MSA and PD. Combined detection of electrophysiological indicators may have higher sensitivity and specificity for differential diagnosis of MSA and PD. The abnormal rates of SSR and RRIV in MSA group and PD group were both high, suggesting that SSR and RRIV may have high sensitivity and low specificity in reflecting autonomic dysfunction and that they may have low value in the differential diagnosis of MSA and PD.

## Data Availability

The data that support the findings of this study are not publicly available due to local ownership of the data but are available from the corresponding author upon reasonable request.

## Abbreviations

PD: Parkinson’s disease
MSA: Multiple system atrophy
SSR: Sympathetic skin response
RRIV: R-R interval variation
BCR: Bulbocavernosus Reflex
K-S: Kolmogorov–Smirnov
SD: Standard deviation
AUC: Area under the curve
IQR: Interquartile range
ROC: Receiver operating characteristic
